# Anti-Alzheimer’s Potency of Rich Phenylethanoid Glycosides Extract from *Marrubium vulgare* L.: In Vitro and In Silico Studies

**DOI:** 10.3390/ph17101282

**Published:** 2024-09-27

**Authors:** Mahmoud Emam, Samah A. El-Newary, Hanan Y. Aati, Bin Wei, Mohamed Seif, Abeer Y. Ibrahim

**Affiliations:** 1 Phytochemistry and Plant Systematics Department, National Research Centre, Dokki, Giza 12622, Egypt; mahmoudemamhegazy2020@gmail.com; 2 Medicinal and Aromatic Plants Research Department, Pharmaceutical and Drug Industries Research Institute, National Research Centre, 33 El Bohouth St., Dokki, Giza 12622, Egypt; samahelnewary@yahoo.com; 3 Pharmacognosy Department, College of Pharmacy, King Saud University, P.O. Box 22452, Riyadh 11495, Saudi Arabia; hati@ksu.edu.sa; 4 College of Pharmaceutical Science & Collaborative Innovation Center of Yangtze River Delta Region Green Pharmaceuticals, Zhejiang University of Technology, Hangzhou 310014, China; binwei@zjut.edu.cn; 5 Food Toxicology and Contaminants Department, Food Industries and Nutrition Research Institute, National Research Centre, Dokki, Giza 12622, Egypt

**Keywords:** anti-inflammatory, anti-cholinesterase, antioxidant, anti-tyrosinase, *Marrubium vulgare*, molecular docking

## Abstract

Background/Objectives: *Marrubium vulgare* L. (*M*. *vulgare*), the white horehound, is well known for treating inflammation-related diseases. Methods: In this context, we investigated the efficacy of *M. vulgare* ingredients in treating Alzheimer’s disease using various in vitro and in silico antioxidant, anti-inflammatory, anti-cholinesterase, and anti-tyrosinase mechanisms. Results: In our results, sixty-one components were tentatively identified using gas and liquid chromatography (GC-MS and LC-MS^n^) and categorized as hydrocarbons, fatty acids, and polyphenolics. The extract inhibited linoleic oxidation with an IC_50_ value of 114.72 µg/mL, captured iron (Fe^2+^) ions with an IC_50_ value of 164.19 µg/mL, and displayed reducing power. In addition, the extract showed radical-scavenging ability towards DPPH^•^, NO^•^, ABTS^•+^, and H_2_O_2_ assays compared to L-ascorbic acid and butylated hydroxytoluene. The DPPH^•^ was scavenged by 77.62% at 100 µg/mL, and NO^•^, ABTS^•+^, and H_2_O_2_ were scavenged with IC_50_ values of 531.66, 117.51, and 143.10 µg/mL, respectively. *M. vulgare* also exhibited discriminating anti-inflammatory potency against cyclooxygenase (COX-2) with IC_50_ values of 619.15 µg/mL compared to celecoxib (*p* > 0.05). Notably, three Alzheimer’s biomarkers, acetylcholinesterase (AChE), butyrylcholinesterase (BChE), and tyrosinase were significantly inhibited. The molecular docking study supposed that the phenylethanoid glycosides of samioside and forsythoside B inhibited AChE and tyrosinase enzymes with low binding affinities of −9.969 and −8.804 kcal/mol, respectively. Marruboside was a proper inhibitor of COX and BChE enzymes with a binding score of −10.218 and −10.306 kcal/mol, respectively. Conclusions: *M. vulgare* extract showed significant inhibitory actions, which suggest that it could have a promising potential as an anti-Alzheimer agent.

## 1. Introduction

*Marrubium vulgare* L. (*M. vulgare*) (Family *Lamiaceae*), is a grey-leaved non-woody perennial plant with stems that belongs to the *Lamiaceae* family. *M. vulgare* is commonly known as white horehound or common horehound. It grows in warm climates, including Europe, North Africa, and Central Asia. The plant is tall and typically reaches 1.5–2.4 m. It has hairy roots, multiple stems, long oval leaves, and white flowers [1].

*Marrubium* is a Latin word derived from the Hebrew word marrob, which means bitter juice, and vulgare meaning ‘common’ or ‘well known’. The English phrase ‘horehound’ is derived from the Old English terms har and hune, which mean a downy plant [1]. White horehound has been linked to folk medicine from at least the first century BC, when it was mentioned as a remedy for respiratory issues in the treatise *De Medicina* by Roman encyclopedist Aulus Cornelius Celsus [2]. Also, it is recommended to treat the teeth, digestion, skin problems, boils, rheumatism, inflammation, sore eyes, hypertension, hyperglycemia, and night blindness. In addition, its volatile oil is a relaxant and can be used for heart palpitations [3].

In recent years, several studies have confirmed the remarkable pharmacological activities of *M. vulgare* both in vivo and in vitro. The plant exhibited noteworthy analgesic activity, anti-edematogenic activity, anti-inflammatory activity, sedative activity, antidiabetic activity, anti-spasmodic activity, anti-ulcer effects, hypolipemic activity, anti-hypertensive action, a hepatoprotective effect, immunomodulatory activity, antimicrobial and anti-*Helicobacter pylori* effects, antiviral activity, and is anti-tumorigenic against human colorectal cancer cells [1,3,4,5,6,7]. Also, different *M. vulgare* extracts showed antiparasitic activity, antiprotozoal activity, antiplasmodial activity, and anthelmintic activity [1], and can be a significant source of natural herbicides to control weeds in crop fields [8]. These pharmacological activities were attributed to the presence of many interesting metabolites that have been isolated from *M. vulgare* as an essential oil, terpenes, flavonoids, coumarins, phenolic acids, and phenylpropanoids [1].

Globally, *M. vulgare* is described in German Commission E publications as a cold remedy, digestive, and choleretic. The plant produces the popular horehound candy, which is said to alleviate coughs, hoarseness, and bronchitis thanks to its pleasant flavor. It is generally deemed safe in the United States and is widely used as a flavoring agent. Currently, some *M. vulgare* herbal medicinal formulations are used as an expectorant in cold-related coughs, and for symptomatic treatment of minor dyspeptic issues such as bloating and gas and a brief lack of appetite [9]. Even though there are just 33 licensed pharmaceutical formulations containing white horehound in India, it is well-known in the United States [5]. In 2015, *M. vulgare* formulations were the best-selling herbal dietary supplements, generating around USD 106 million in commercial sales [10].

For millennia, medicinal and aroma plants, particularly those having ethnopharmacological applications, have been used as natural sources of treatments and healthcare against contemporary problems [11]. One of these is the prevalent type of dementia known as Alzheimer’s disease (AD), which accounts for at least two-thirds of cases in individuals aged 65 and older [12].

Alzheimer’s disease (AD) is disorder that is a degenerative defect, which leads to brain cell damage and eventual cell death. Early symptoms of the disease include short-term memory loss and impaired conversation skills. Late-stage symptoms include significant memory loss and difficulties in performing simple daily tasks [13]. Currently, there is no cure for Alzheimer’s, but existing medications can improve brain function in some patients, allowing them to remain independent for many years. Nevertheless, these drugs are associated with severe side effects [14]. Therefore, it is essential to search for new remedies with fewer side effects. Several intriguing natural products for Alzheimer’s have been extracted from medicinal plants like *Balanites aegyptiaca* dates’ hydroethanolic extract [15], *Calendula Suffruticosa* Vahlsubsp. boissieri Lanza [16], and *Olea dioica* [17].

In addition, tyrosinase is the enzyme that catalyzes L-tyrosine to convert to 3,4-dihydroxyphenylalanine (DOPA). L-Dopa is the precursor to dopamine, norepinephrine (noradrenaline), and epinephrine (adrenaline). Dopamine plays numerous essential roles in the brain and body [18]. On the other hand, tyrosinase can impair neurons by generating dopamine quinones, highly reactive and toxic species, through the oxidation of the catechol ring of dopamine. Dopamine quinones may bind to the sulfhydryl active functional groups of cysteine amino acids in the cytosol, forming protein adducts that can irreversibly alter or decrease protein activity. Dopamine oxidation also affects the dopamine transporter, glutamate transport, and mitochondrial respiration. Cytosolic quinones can also reach the nucleus and cause covalent DNA changes [19]. Tyrosinase activity also increases the induction of H_2_O_2_, acting as an enhancer of cellular oxidative stress [20]. Greggio et. al. [19] concluded that tyrosinase may be damaging to catecholaminergic neurons by raising the concentration of dopamine-derived reactive species during oxidative stress situations. Tyrosinase blockers are a class of medicines that obstruct tyrosinase action. Even though there are numerous tyrosinase inhibitors available, only some are currently promoted as “safe”. Therefore, the quest for novel tyrosinase blockers is crucial [21]. Tyrosinase blockers derived from natural sources have sparked widespread interest among researchers due to their decreased toxicity and improved bioavailability. Microorganisms and plants have steadily emerged as exploration hotspots for tyrosinase inhibitor extraction. Some plant-derived bioactive components limit tyrosinase potency, although at higher doses than recognized blockers such as pyran-4-one derivative (kojic acid), nonanedioic acid (azelaic acid), and hydroquinone-*β*-D-glucopyranoside (arbutin) [22].

As well, acetylcholine (ACh) functions as a neurotransmitter in the human brain and body, sending signals to other cells such as neurons, muscle cells, and gland cells. ACh regulates memory by influencing the acquisition, encoding, consolidation, reconsolidation, extinction, and recovery of memory [23]. The cholinesterase (ChE) enzyme, either acetylcholinesterase or butyrylcholinesterase, aids in its breakdown to choline and acetate at cholinergic synapses. Unfortunately, the brains of individuals with Alzheimer’s disease have lower levels of acetylcholine compared to healthy individuals, which is associated with increased AChE activity [24]. Thus, inhibiting AChE preserves acetylcholine levels and improves cholinergic function. Consequently, AChE inhibitors are often considered an ideal treatment for Alzheimer’s syndrome. AChE inhibitors are indeed the most effective treatments for the cognitive symptoms of Alzheimer’s syndrome, senility, dyskinesia, and paralysis agitans [25,26].

The current investigation sought to identify new sources of active medicines from *M. vulgare* aerial parts extract to prevent or treat Alzheimer’s syndrome and illustrate their possible mechanistic action. In addition, its effect on neurotransmitter degradation and tyrosinase suppression was investigated. Also, its anti-inflammatory and antioxidant potencies were examined in vitro and further explained with molecular docking studies. This study introduces *M. vulgare*’s phenylethanoid glycoside metabolites as a viable adjuvant therapy for Alzheimer’s disease.

## 2. Results

### 2.1. Phytochemical Composition of the M. vulgare Extract

Preliminary assays of the *M. vulgare* extract revealed the presence of 61.96 ± 5.15 mg/g of alkaloids, 240.46 ± 12.19 mg of Gallic acid/g of polyphenols, and 100.95 ± 3.77 mg/g of tannins in the extract.

Also, the nonpolar constituents of *M. vulgare* extract were introduced into GC-MS analysis after different chemical treatments to bring the metabolites to a suitable form of derivatization for analysis (Table 1 and Table 2). The lipoidal matter (USM) was analyzed using GC-MS analysis after the silylation process was carried out (Table 1). It was ascertained that steroidal structures represent 93.56% of the total identified components, while oxygenated hydrocarbons and sesquiterpenes represent 2.42% and 2.6%, respectively. Traces of polyunsaturated fatty acids were liberated into the solution after silylation and detected at 1.42%.

On the other hand, Table 2 illustrates the relative concentration percentage of saturated and unsaturated fatty acid methyl esters as 2.59% and 1.62%, respectively. In addition, benzoic acid derivatives were the major identified saponified matter in *M. vulgare*, accounting for 95.5%.

In addition, the polar constituents of *M. vulgare* extract were fractionated into butanol and aqueous fractions before injection in the LC/MS^n^. Thirty-seven metabolites were identified based on their molar masses, and their fragment ions belong to phenolic acids, flavonoid glycosides, aglycones, a diterpenoid lactone, phenylethanoid glycoside, and flavonoid (*O* and *C*) high glycosides (Table 3 and Table 4).

**Figure 1:**
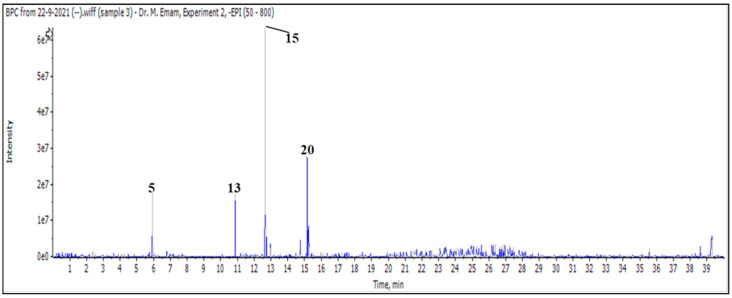
LC/MS2 base peak chromatogram (BPC) of *M. vulgare* extract for butanol fraction.

After injecting a butanol sample, 26 molecules were identified as phenolic acids, aglycones, and mono glycosides (Figure 1 and Table 3).

**Figure 2:**
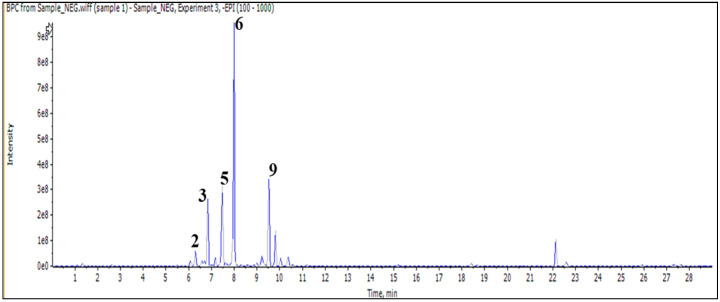
LC/MS^2^ base peak chromatogram (BPC) of *M. vulgare* extract for aqueous fraction.

In addition, the injection of an aqueous sample led to the identification of 11 compounds classified as phenylethanoid glycoside, flavonoid (*O* and *C*) high glycoside, and a diterpenoid lactone (Figure 2 and Table 3).

### 2.2. Biological Activities of the M. vulgare Extract

#### 2.2.1. Antioxidant Potencies of the *M. Vulgare* Extract

##### Inhibition of Lipid Peroxidation

**Figure 3:**
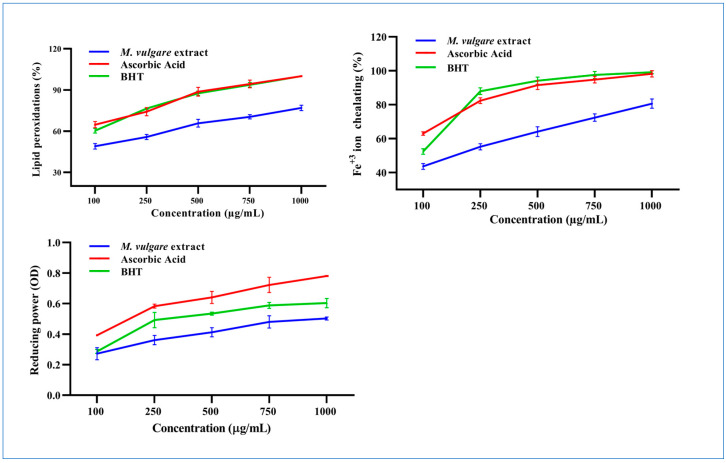
The *M. vulgare* extract and reference materials, ascorbic acid and butylated hydroxytoluene (BHT), were tested at various concentrations (100–1000 μg/mL) for lipid peroxidation inhibition, Fe^+2^ ion chelation capacity, and reducing power capability. The data are displayed as average ± SE (*n* = 3, *p* < 0.05).

At 100 µg/mL, *M. vulgare* extract inhibited linoleic oxidation by 46.89% ± 1.98, which rose to 76.91% ± 1.95 at a concentration of 1000 µg/mL with an IC_50_ value of 114.72 µg/mL compared to the 28.64 and 39.30 µg/mL of ascorbic acid and BHT, respectively (Figure 3).

##### Fe^2+^ Chelation Ability

The Fe^2+^ chelation ability of *M. vulgare* was determined based on its ability to prevent the formation of the Fe^2+^–ferrozine complex. At a concentration of 100 µg/mL, the *M. vulgare* extract captured 43.62% ± 1.70 of the Fe^2+^ ions from the reaction medium, which increased to 80.64% ± 2.70 at a concentration of 1000 µg/mL with an IC_50_ value of 164.91 µg/mL. This was compared to 26.36 and 68.88 µg/mL for ascorbic acid and BHT, respectively (Figure 3).

##### Reducing Power of *M. vulgare*

The reducing power of *M. vulgare* was indicated by the transformation of Fe^3+^ ions to Fe^2+^. The Fe^3+^ ion reduction capacity of the extract was estimated by absorbance measurements. Collectively, the *M. vulgare* extract exhibited relatively moderate reducing power compared with ascorbic acid and BHT (Figure 3).

#### 2.2.2. Scavenging Activity

##### DPPH^•^ Scavenging Activity

**Figure 4:**
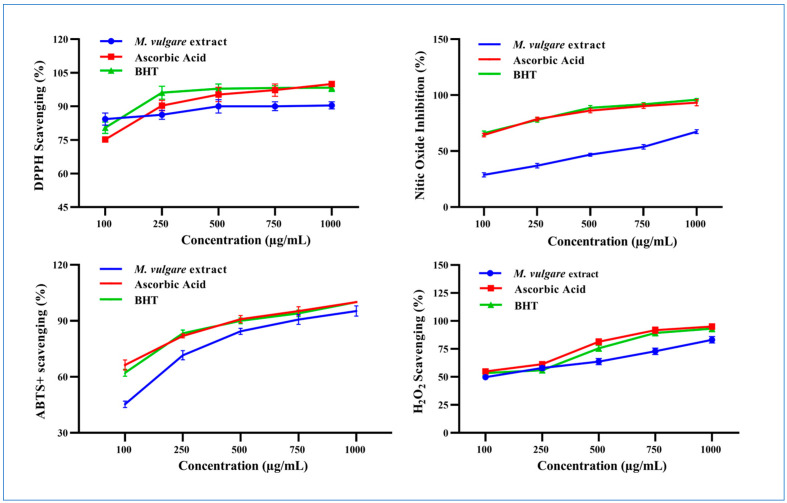
The scavenging properties of *M. vulgare* extract and reference materials including ascorbic acid and BHT at various doses (100–1000 μg/mL) for DPPH^•^, NO^•^ scavenging, ABTS^•+^, and H_2_O_2_. BHT: butylated hydroxytoluene, DPPH: 1,1 diphenyl-2-picryl-hydrazyl free radical, ABTS^+^: 2,2-azinobis (3-ethylbenzthiazoline-6-sulfonic acid), NO: nitric oxide, and H_2_O_2_: hydrogen peroxide. The statistics are presented as average ± SD. The data were analyzed using an independent *t*-test (*n* = 3, *p* < 0.05).

*M. vulgare* extract displayed potent DPPH^•^ scavenging activity and neutralized the radical to the stable form with a percent inhibition of 77.62% ± 2.00 at 100 µg/mL, which increased to 90.44% ± 1.56 at 1000 µg/mL (Figure 4).

##### NO^•^ Scavenging Activity

*M. vulgare* extract inhibited the liberation of NO^•^ from the SNP reaction in a concentration-dependent manner with an IC_50_ value of 531.66 µg/mL (Figure 4). Approximately 28.79% ± 1.80 of NO^•^ was captured in a reaction medium containing 100 μg/mL of *M. vulgare* extract, compared to ascorbic acid and BHT (64.39% ± 1.61 and 65.99% ± 2.00, respectively). NO^•^ scavenging increased to 67.35% ± 1.65 by increasing the concentration of the *M. vulgare* extract to 1000 µg/mL compared to the 93.27% ± 2.73 and 95.88% ± 1.89 of ascorbic acid and BHT, respectively.

##### ABTS^•+^ Scavenging Power

The *M. vulgare* extract exhibited ABTS radical cation-scavenging ability with an IC_50_ value of 117.51 μg/mL compared to L-ascorbic acid and BHT (Figure 4). At 100 μg/mL, the extract neutralized 45.28% ± 1.72 of the ABTS radical cations in the reaction medium. Moreover, increasing the concentration of the extract to 1000 μg/mL enhanced the neutralization to 95.51% ± 2.74, close to the scavenging activity recorded for L-ascorbic acid and BHT (i.e., 100% ± 0.00).

##### H_2_O_2_ Scavenging Activity

At 100 μg/mL, *M. vulgare* extract reduced H_2_O_2_ molecules in the medium by 49.73% ± 2.14 compared to the 54.92% ± 1.95 and 53.47% ± 2.53 of L-ascorbic acid and BHT, respectively (Figure 4). By increasing the extract concentration to 1000 μg/mL, the percentage of scavenged H_2_O_2_ molecules increased to 83.16% ± 2.24 compared to the 95.00% ± 2.50 and 93.16% ±2.84 of L-ascorbic acid and BHT, respectively. *M. vulgare* extract reduced H_2_O_2_ with an IC_50_ value of 143.10 μg/mL compared to the 91.92 and 129.98 μg/mL of L-ascorbic acid and BHT, respectively.

#### 2.2.3. Anti-Inflammatory Effects of the Crude *M. vulgare* Extract

##### COX-1 Inhibition

**Figure 5:**
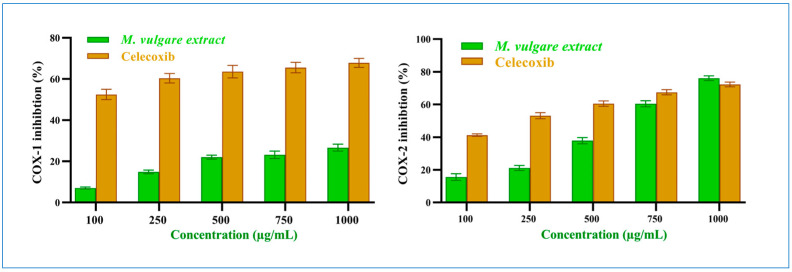
*M. vulgare* extract and celecoxib at varying dosages (100–1000 μg/mL) inhibit COX-1 and COX-2 enzymes. Statistics provided as average ± SD (*n* = 3, *p* < 0.05).

*M. vulgare* extract had an insignificant inhibitory result on COX-1 (*p* > 0.05) compared to celecoxib. At 100 μg/mL, *M. vulgare* suppressed COX-1 activity by 7.04% ± 0.50, which increased to 26.64% ± 1.70 at 1000 μg/mL (Figure 5), with an IC_50_ value of 2412.25 μg/mL compared to the 64.22 μg/mL of celecoxib.

##### COX-2 Inhibition

On the other hand, *M. vulgare* extract exhibited potent inhibitory effects towards COX-2 (Figure 5) with an IC_50_ value of 619.15 μg/mL, compared to the 221.72 μg/mL of celecoxib. It showed moderate inhibition with 15.59% ± 2.09 at 100 μg/mL, which increased to 76.07% ± 1.41 at 1000 μg/mL. Compared to celecoxib, 41.23% ± 0.77 and 72.49% ± 1.51 inhibitions were established at the same concentrations with *p* > 0.05. Notably, the percentage of COX-2 inhibition at a high extract concentration of 750 and 1000 μg/mL was similar to that of celecoxib. Based on the IC_50_ values of the extract, it was determined that the crude extract of *M. vulgare* has specific anti-inflammatory actions against COX-2.

#### 2.2.4. Effect of *M. vulgare* on the Alzheimer’s Syndrome Biological Markers

The anti-Alzheimer’s syndrome activities of *M. vulgare* were investigated by elucidating its role in preventing neurotransmitter degradation and the inhibiting of neurofibrillary tangle formation.

##### Inhibition of Neurotransmitter Degradation

1. Acetylcholinesterase Inhibition

**Figure 6:**
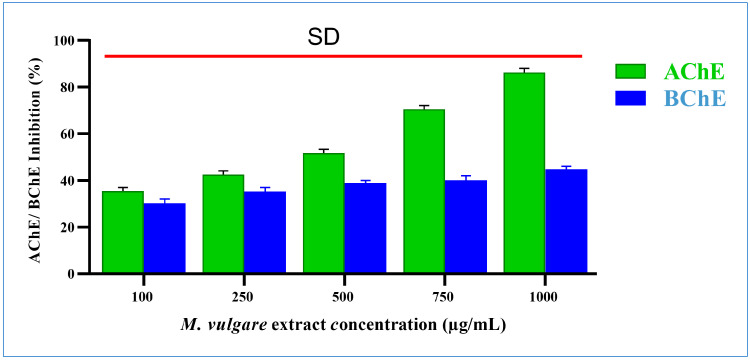
*M. vulgare* extract was tested at various concentrations (100–1000 μg/mL) for anti-cholinesterase activity. Statistics shown as average ± SD (*n* = 3), *p* < 0.05.

*M. vulgare* extract significantly inhibited acetylcholinesterase by 35.41% ± 1.54 and 86.24% ± 1.76 at doses of 100 and 1000 µg/mL, respectively (Figure 6), with an IC_50_ value of 321.16 µg/mL.

2. Butyrylcholinesterase Inhibition

The *M. vulgare* extract inhibited butyrylcholinesterase activity in a dose-dependent manner with an IC_50_ value of 2195.35 µg/mL. At 100 µg/mL, butyrylcholinesterase activity was decreased by 30.24% ± 1.76. The percentage of inhibition grew progressively as the concentration of the extract increased. The highest inhibition, with a value of 44.76% ± 1.24%, was recorded at 1000 µg/mL (Figure 6).

##### Inhibition of Tyrosine (Neurofibrillary Tangle Formation)

**Figure 7:**
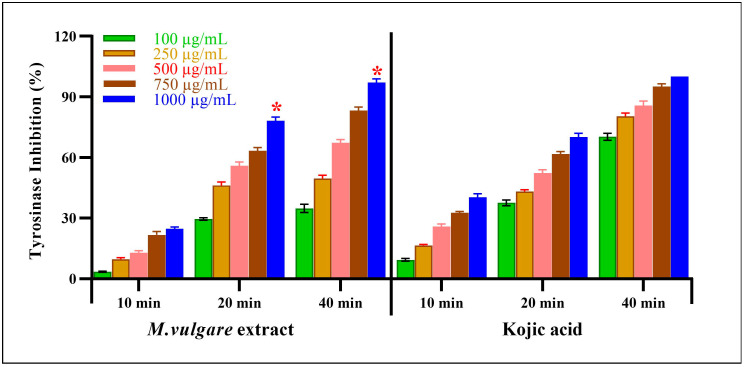
*M. vulgare* extract was examined for anti-tyrosinase activity at varying doses (100–1000 μg/mL). Statistics are presented as average ± SD (*n* = 3, *p* < 0.05). Statistics are marked with (*), indicating a remarkable change with kojic acid.

The *M. vulgare* extract also showed inhibitory tyrosinase effects, which were enhanced by increasing the incubation time and extract concentration (Figure 7). At the lowest extract concentration of 100 μg/mL, tyrosinase was inhibited by 3.54% ± 0.22 after incubation for 10 min. After 20 min of incubation, the inhibition percentage reached 29.58% ± 0.66, whereas, after 40 min, a 34.78% ± 2.05 inhibition was achieved. At the same concentration and incubation times, kojic acid resulted in 9.34% ± 0.66, 37.55% ± 1.45, and 70.32% ± 1.68 inhibitions, respectively. 

Notably, at the highest concentration of the *M. vulgare* extract, i.e., at 1000 μg/mL, the inhibitory tyrosinase activity increased to 24.73% ± 0.84, 78.15% ± 1.85, and 97.14% ± 1.86 after 10, 20, and 40 min of incubation, respectively. In the case of kojic acid, at the same concentration and incubation times, 40.35% ± 1.65, 70.23% ± 1.77, and 100.00% ± 0.00 inhibition was achieved. At different incubation times of 10, 20, and 40 min, *M. vulgare* extract had lower IC_50_ values of 2.15, 23.05, and 12.19 μg/mL compared to the kojic acid IC_50_ values of 6.00, 29.62, and 62.97 μg/mL, respectively.

### 2.3. Molecular Docking Simulation

**Figure 8:**
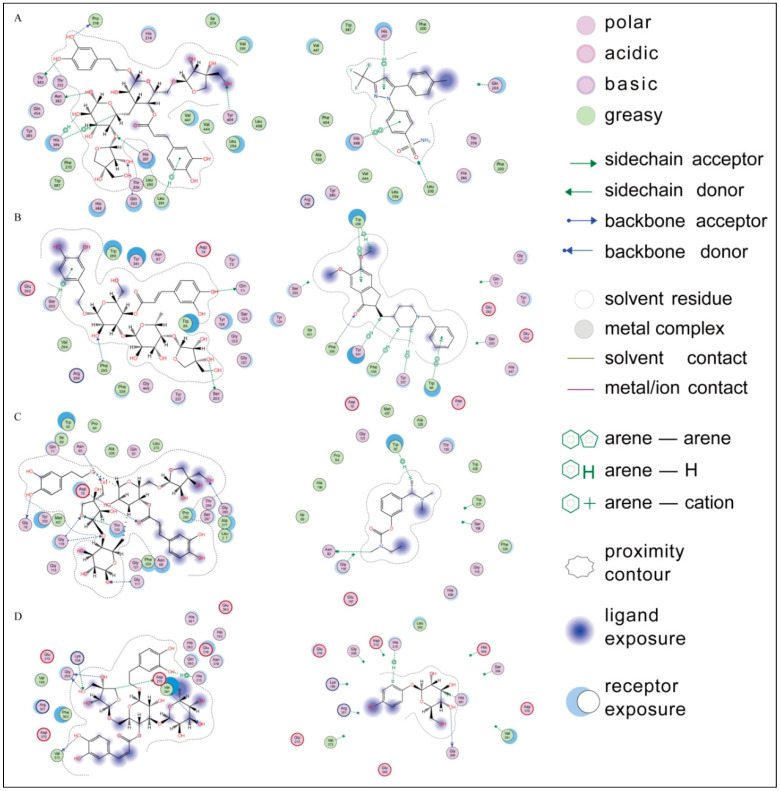
Ligand interactions of (**A**) marruboside and celecoxib, (**B**) samioside and Donepezil, (**C**) marruboside and Rivastigmine, and (**D**) forsythoside and arbutin with the active sites of cyclooxygenase (COX-2), acetylcholinesterase, butyrylcholinesterase, and tyrosinase, respectively.

A molecular docking analysis was conducted to understand the interactions between the compounds and the target enzymes and predict the potential binding sites and binding modes, providing theoretical support for the experimental results. As shown in Supplementary Table S1, all the compounds demonstrated varying degrees of binding affinities to the active sites of cyclooxygenase (COX-2), acetylcholinesterase, butyrylcholinesterase, and tyrosinase. Notably, marruboside, samioside, and forsythoside B exhibited the lowest binding affinities, suggesting their potential as effective inhibitors in crude extract samples. To contextualize these findings, we compared the binding affinities and interaction patterns of these compounds with well-known inhibitors of the respective enzymes (Figure 8). 

For COX-2, marruboside showed significant interactions, forming four hydrogen bonds with His^207^, Asn^382^, Thr^383^, and Tyr^409^, along with three H-π interactions with His^386^ and Leu^391^. These interactions are comparable to those observed with selective COX-2 inhibitors such as celecoxib and Meloxicam, which are FDA-approved nonsteroidal anti-inflammatory drugs (NSAIDs) known for their improved gastric safety. Celecoxib forms H-π interactions with His^207^, while Meloxicam forms hydrogen bonds with His^207^ and His^386^ in the active site. This suggests that marruboside may exhibit inhibitory effects similar to these established COX-2 inhibitors.

Similarly, Donepezil and Rivastigmine are potent, selective, non-competitive, and rapidly reversible inhibitors of both acetylcholinesterase and butyrylcholinesterase, and are approved for the treatment of Alzheimer’s disease. Therefore, these two drugs were used as reference compounds. Samioside demonstrated robust docking results with acetylcholinesterase, forming hydrogen bonds with Gln^71^, Ser^203^, Ser^293^, and Phe^295^, as well as hydrophobic interactions with Trp^86^, Trp^286^, and Tyr^341^. Donepezil also forms hydrophobic interactions with Trp286 and Tyr^341^, while Rivastigmine forms hydrogen bonds with Phe^295^ and hydrophobic interactions with Trp^286^. This comparison indicates that samioside could potentially serve as an effective acetylcholinesterase inhibitor.

Marruboside also exhibited strong affinity for the active site of butyrylcholinesterase, forming multiple interactions with key residues such as Asp^70^, Gly^78^, Asn^83^, Gly^116^, Gly^117^, Thr^120^, and Gly^283^. Donepezil forms hydrogen bonds with Gly^116^, while Rivastigmine forms hydrogen bonds with Asn^83^. Therefore, marruboside may act as a dual inhibitor for COX-2 and butyrylcholinesterase. Finally, forsythoside B primarily interacted with tyrosinase through an H-π interaction with His^215^ and six hydrogen bonds with Lys^198^, Gly^209^, Asp^212^, Val^373^, and Val^393^. This interaction pattern is similar to that of arbutin, which forms an H-π interaction with His^215^. Thus, forsythoside B could have potential applications in inhibiting tyrosinase activity.

The docking results not only highlight the potential inhibitory effects of marruboside, samioside, and forsythoside B but also provide a mechanistic understanding of how these compounds might exert their biological activity. Comparing these results with known inhibitors further validates the potential of these compounds as leads for drug development, underscoring the importance of molecular docking in identifying promising anti-Alzheimer agents.

## 3. Discussion

Many plant extracts and phytochemical compounds have been found to exert anti-Alzheimer’s activities [32,33,34,35]. In the current study, the crude extract of *M. vulgare* displayed potent antioxidant capability and selective anti-inflammatory effects against COX-2, as well as a significant inhibition of cholinesterase and tyrosinase.

Previously, Neganova et al. [36] found a correlation between Fe^2+^ binding activity and the presence of a free nitrogen atom in the modified alkaloid derivatives. Also, a remarkable relationship was found between antioxidant power and the total polyphenol content, which can eliminate radicals to form stable radical intermediates due to hydrogen-donating ability [37]. In addition, flavonoids show unique antioxidant activities, including the inhibition of oxidant generation, preventing oxidants from attacking cells, eliminating oxidative stress, and supporting the cell’s antioxidant defense. The free radical-scavenging ability of flavonoids results from rapid hydrogen donation to radical species. Flavonoids inhibit the lipid peroxidation and Fe^2+^-catalyzed oxidation of glutamine synthase through free radical scavenging and metal ions from the catalytic sites via chelation [38].

In the current study, the total alkaloid and phenolic contents of the *M. vulgare* extract were 6.20% and 24.05%, which explained the antioxidant capability of the *M. vulgare* extract.

Moreover, the discriminating anti-inflammatory capabilities of the *M. vulgare* extract discovered in this study may be related to the extract’s antioxidant and anti-inflammatory potencies, particularly its NO-scavenging capacity. Under normal physiological conditions, NO is an anti-inflammatory mediator; however, an excessive production of NO promotes inflammation. The inducible nitric oxide synthase enzyme (iNOS) catalyzes the synthesis of NO in biological tissues [39]. Moreover, the up-regulation of COX-2 in endothelial cells is often accompanied by an elevation in iNOS levels. Thus, NO inhibition is considered an important therapeutic strategy for controlling inflammatory diseases [40]. The current findings displayed that *M. vulgare* extract succeeded in inhibiting NO compared to the tested standard.

In addition, acetylcholinesterase is the enzyme responsible for the breakdown of acetylcholine into choline and acetate. Inhibiting the activity of this enzyme is considered advantageous in the management of Alzheimer’s disease. Alzheimer’s disease is characterized by low acetylcholine levels in the brain and myasthenia gravis. Hence, utilizing acetylcholinesterase inhibitors is the most effective approach to treating the cognitive disorders associated with Alzheimer’s disease [40]. Galantamine, donepezil, rivastigmine, and memantine are natural product derivatives commonly used to treat Alzheimer’s disease as acetylcholinesterase inhibitors [26] for one of two binding sites. The first binding site interacts with positively charged nitrogen atoms in alkaloids, while the second interacts with non-alkaloid components like phenols [41].

So, the inhibitory acetylcholinesterase effects of the *M. vulgare* extract demonstrated in this study were consistent with the outcomes previously reported by Schlemper et al. [42]. They attributed the anti-spasmodic activity of the extract to its inhibitory effects on neurotransmitters, including acetylcholine, prostaglandin E, histamine, bradykinin, and oxytocin, with advanced selectivity for cholinergic levels. The evident anti-acetylcholinesterase and anti-butyrylcholinesterase effects of the *M. vulgare* extract obtained in the current study were attributed to its alkaloid, phenol, and flavonoid content. The hydroxyl (OH) groups on the aromatic ring (B-ring) of flavonoids react with the amino acid residues in the enzyme’s peripheral anionic site, blocking the active site [43]. Increasing the number of OH or/and OCH_3_ groups on the phenol ring enhances the inhibitory effects on acetylcholinesterase (e.g., cinnamic acid and derivatives). Additionally, the presence of a propenoic (CH=CHCOOH) group as caffeic acid increases the anti-acetylcholinesterase effects [44].

Our results were in agreement with those published before by Neagu et al. [45] that reported how the hydroxycinnamic acid, rutin, and caffeic acid of *Centarium umbellatum* and *Pulmonaria officinalis* exhibited anti-acetylcholinesterase activities [46]. Moreover, trihydroxybenzoic acid and pentahydroxyflavone exhibited anti-acetylcholinesterase and anti-butyrylcholinesterase effects, while 5-caffeoylquinic acid and naringenin displayed anti-butyrylcholinesterase activity [47]. 4-hydroxy-3-methoxycinnamic acid isolated from the ethyl acetate extract of *Impatiens bicolor* Linn showed significant anti-acetylcholinesterase effects [48]. Also, Heoa et al. [49] reported that naringenin isolated from *Citrus junos* exhibits inhibitory acetylcholinesterase activity. It was also demonstrated that coumarin interacts with the catalytic sites of the enzymes via the rings at the 3- and 4-positions [50], resulting in the effective inhibition of acetylcholinesterase and butyrylcholinesterase. By contrast, most recognized blockers have nitrogen atoms such as the isolated galantamine alkaloid structure from *Galanthus nivalis* that inhibit acetylcholinesterase through their complex nitrogen-containing configurations [51].

Another, tyrosinase is a Cu-based oxidase found in fauna and flora tissues. It is the enzyme that limits the pace of melanin formation. Tyrosinase is also involved in the synthesis of neuromelanin and the neuronal damage associated with Parkinson’s disease [46]. L-Dopa, a precursor to dopamine, is created when tyrosine hydroxylase acts on tyrosine. In Alzheimer’s syndrome, the accumulation of the A-peptide causes the formation of amyloid plaque, which is monitored by neurodegenerative alterations. Previous research has shown that L-Dopa and dopamine liquefy A-peptide fibrils and reduce protein tangle formation. Furthermore, Storga et al. [51] demonstrated the dopaminergic deficit in Alzheimer’s disease brain samples [52]. 

The analyzed flora extracts inhibited tyrosinase activity, albeit at higher doses than recognized blockers such as kojic acid, azelaic acid, and arbutin [53]. Numerous studies have been conducted to regulate tyrosinase activity utilizing metal chelators such as 5-Hydroxy-2-(hydroxymethyl)-4-pyrone, 1,2-dihydroxybenzene, 2,5-dihydroxybenzoic acid, flavonol, and hydroxamic acid, taking into account the copper structure of tyrosinase [54]. In general, flavonoids containing a hydroxyl (-OH) moiety at the A and B rings are potent tyrosinase blockers. Their mechanism of action involves Cu^2+^ chelate formation [55]. In addition, quercetin and kaempferol have also been shown to interfere with tyrosinase activity through the chelation of copper in the enzyme’s active site [21]. Hence, the iron (II)-chelating activity of *M. vulgare* is of great significance, because it has been proposed as anti-tyrosinase activity, preventing the metal ions’ transition that can contribute to oxidative damage in neurodegenerative disorders like Alzheimer’s and Parkinson’s disease [56]. These results explain the anti-tyrosinase action of the *M. vulgare* phytochemical constituents.

Among the phytochemical constituents identified in *M. vulgare* extract, chrysin is reported to improve memory in the aging brain by slowing the growth of reactive species and also to protect hippocampal neurons from injury and restore memory deficits in mice with chronic cerebral hypoperfusion. Chrysin can reduce apoptosis and memory problems associated with traumatic brain damage [57]. Apigenin ameliorates AD-associated memory impairment, reduces the Aβ plaque burden, and inhibits oxidative stress [58]. Luteolin has potential benefits for the central nervous system, including reduced microglia activation, neuronal damage, and high antioxidant activity. In vivo studies on rat models have shown that tetrahydroxyflavone protects against the cognitive impairment caused by prolonged cerebral hypoperfusion. Luteolin’s antioxidant activity also reduces the Zn-induced hyperphosphorylation of the protein τ in SH-SY5Y cells and protects obese mice from cognitive impairments due to a high-fat diet [59]. Vanillin can reduce ROS levels and metalloproteinase-9 expression in LPS-stimulated macrophages, suggesting its potential in defending against neurodegeneration and oxidative stress [60]. D-(-)-Quinic acid can prevent aluminum-induced memory impairment by inhibiting AChE activity, promoting DNA repair, and inhibiting NF-κB [61]. In an in vitro model of ischemia, hydroxycinnamic acid derivatives enhanced synaptic transmission recovery following re-oxygenation after oxygen–glucose deprivation and reduced oxidative stress-related cell damage in cell culture models [62]. Additionally, phenylethanoid glycosides (PhGs) reduced neuroinflammation through neuroprotective and anti-inflammatory properties, reducing neuroinflammation and cognitive impairment in transgenic models of Alzheimer’s disease [63]. According to the docking results and the previous literature, the anti-Alzheimer’s efficacy of *M. vulgare* extract could be mediated by PhGs [64,65].

Finally, we conclude that the active ingredients identified by LC/MS in the hydroethanolic extract of *M. vulgare* fought Alzheimer’s through several mechanisms, including (i) suppression of AChE overexpression that prevents ACh hydrolysis, (ii) inhibition of tyrosinase, (iii) suppression of oxidative stress, and (iv) the inhibition of COX-2 and activation of COX-1.

## 4. Materials and Methods

### 4.1. M. vulgare Assemblage and Extract Preparations

**Figure 9:**
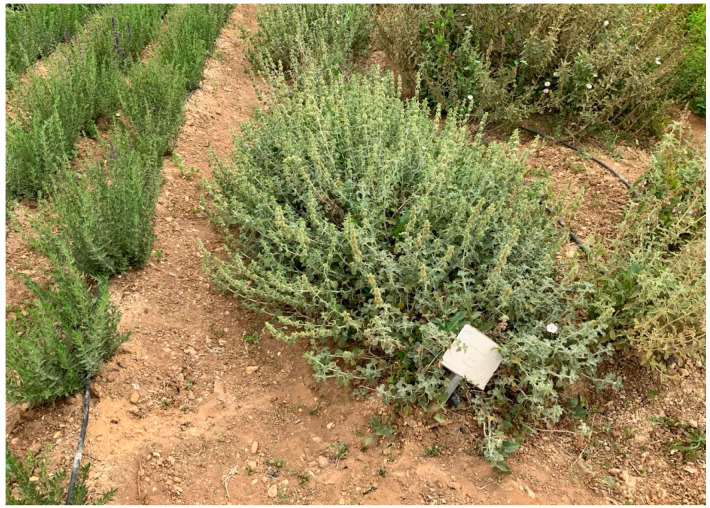
*M. vulgare* plant (Tahaweyah, Bilbeis, Al-Sharqia Governorate 7048302).

*M. vulgare* L. aboveground parts were obtained from the organic farm of Heliopolis University in July 2019 (Tahaweyah, Bilbeis, Al-Sharqia Governorate 7048302, Egypt) (Figure 9). The plants were validated by Prof. Dr. Kamal Zaied, Plant Department, Faculty of Science, Cairo University, Egypt. At room temperature, the aerial segments were dried in the shade, and the powder (1 kg) was macerated with solvent (70% ethanol) for 21 days. The filtrates were mixed and then dried to yield 130 g powder extract using Rotavapor^®^ (Heizbad Hei-VAP, Heidolph, Schwabach, Germany). The lyophilized extract (Christ, Osterode am Harz, Germany) was deposited at −20 °C.

### 4.2. Phytochemical Analysis

#### 4.2.1. Preliminary Chemical Composition of the *M. vulgare* Extract

The total amount of phenolics and tannins was determined using the Folin–Ciocalteu technique [65] and a standard Broadhurst and Jones method [66], respectively. In addition, the alkaloids were determined by Onwuka’s gravimetric method [67].

#### 4.2.2. GCMS Analysis

The nonpolar components of unsaponifiable matter (USM) and saponifiable matter (SM) were extracted, derivatized, and analyzed using Agilent GC-MS technology systems. Wiley and NIST Mass Spectral Libraries data were used for identification.

##### Unsaponifiable Matter (USM) 

*M. vulgare*’s *n*-hexane fraction (0.657 g) was saponified under reflux with 10 mL of 10% potassium hydroxide (KOH) and 4 mL of toluene for 24 h to guarantee full hydrolysis [68]. The saponified solution was concentrated using a rotary evaporator and suspended in 100 mL of distilled water, then extracted with peroxide-free diethyl ether (50 mL × 3) [69]. The mixed ethereal extracts were collected, rinsed with distilled water to remove alkalinity, dried on anhydrous sodium sulfate, and evaporated until dry to yield USM (0.36 g ~ 54.95%).

Bis(trimethylsilyl) trifluoroacetamide (BSTFA)^+^ trimethylchlorosilane (TMCS) (99:1) was used to convert the functional groups of the dried USM sample into trimethylsilyl groups (TMS) [70] before the injection into the GC-MS Agilent technology system that was described previously [71].

##### Saponifiable Matter (SM) Analysis and the Preparation of the Fatty Acid Methyl Esters (FAMEs)

The aqueous alkaline solution that was left after the separation of the USM matter was acidified with diluted hydrochloric acid to liberate the free fatty acids, extracted with peroxide-free diethyl ether (4 × 50 mL), washed with distilled water to eliminate acidity, and dehydrated over anhydrous sodium sulfate [68,72] to yield fatty acid methyl ester (0.295 g~45.05%). The FAME sample was injected into the GCMS system (Agilent Technologies, Santa Clara, CA, USA) as described previously [73].

#### 4.2.3. LC-ESI-MS/MS Analysis

The polar components of the *M. vulgare* sample were analyzed using liquid chromatography–electrospray ionization–tandem mass spectrometry (LC-ESI-MS/MS) (AB Sciex ExionLC AC HPLC coupled with a SCIEX Triple Quad^™^ 5500 LC-MS/MS system) as described previously [74]. 

### 4.3. Biological Potencies of the M. vulgare Extract

#### 4.3.1. Antioxidant Potencies of the *M. vulgare*

The extracted samples and reference materials (i.e., BHT and ascorbic acid) were prepared as methanolic solutions of serial concentrations, specifically 100, 250, 500, 750, and 1000 µg/mL, and were used for all antioxidant determination methods. Antioxidant capacities were spectrophotometrically assayed (Jasco V630, Tokyo, Japan).

##### Lipid Peroxidation-[NH4] SCN (Ammonium Thiocyanate)

The potency of the *M. vulgare* extract to obstruct lipid peroxidation (LP) was assessed according to Duh et al. [75]. An amount of 175 μg Tween-20, 155 μL linoleic acid, and 0.04 mol/L potassium phosphate buffer (pH 7.0) was mixed to make a lineolate emulsion. Samples and standards at different concentrations (1 mL) were added to 4.1 mL linoleate emulsion, 0.02 mol/L phosphate buffer (pH = 7.8), and distilled water (DW), then kept warm in the dark at 40 °C. An amount of 0.1 mL of a mixture of each series was added to 9.7 mL ammonium thiocyanate (75%) and incubated for three minutes, and then 0.1 mL of FeCl_3_ (0.02 mol/L in 3.5% HCl) was added. The peroxide concentration was determined daily at 500 nm. The lipid peroxidation inhibition calculation was designed as the following equation:

*Inhibition* % = ((*A _control_* − *A _sample_*)/*A _control_*) × 100

##### Reduction of Ferric Ions (Fe^3+^)

The ability of the *M. vulgare* extract and the reference compounds to reduce Fe^3+^ ions was described previously [76]. A fixed volume of each tested sample (2.5 mL) was mixed with the same volume of phosphate buffer (0.2 mol/L, pH 6.6) and 1% of K_3_Fe(CN)_6_, and then incubated for 20 min. Subsequently, the same volume of (10%) trichloroacetic acid was introduced to the solution, followed by centrifugation for 10 min at 1000× *g* (MSE Mistral 2000; Sanyo Gallenkamp PLC, Leicestershire, UK, serial no.: S693/02/444). Finally, 2.5 mL of the upper layer was added to 2.5 mL methanol and 0.5 mL ferric chloride (FeCl_3_, 0.1%) and read at 700 nm.

##### Ferrous Ions (Fe^2+^)-Chelating Capacity

The Fe^2+^ chelating capacity of the extract and the standard materials were assayed based on the technique of Dinis et al. (1994) [77]. An amount of 0.05 mL FeCl_2_ (2 mmol/L) was added to each sample. The experiment was initiated by introducing 0.2 mL ferrozine (5 mmol/L), and the mixture was agitated strongly and left to stand at room temperature for 10 min before being measured at 562 nm. The formula provided the percentage of the inhibition of ferrozine-Fe^2+^ complex formation:

*Inhibition* (%) = ((*A _control_* − *A _sample_*)/*A _control_*) × 100

#### 4.3.2. Scavenging Properties

##### NO^•^ Radical-Scavenging Power

The capability of the *M. vulgare* extract and the reference compounds to capture NO• was evaluated based on the method of Marcocci et al. [78]. NO^•^ generated from sodium nitroprusside (SNP) was detected using the Griss mixture, which consisted of 1% sulfanilamide in 5% ortho-H_3_PO_4_ and 0.1% naphthylethylene diamine dihydrochloride. Initially, 2 mL of each sample was added to SNP (10 mmol/L) and then incubated at 25 °C for 150 min. Subsequently, 1 mL of the mixture was diluted with 1 mL of Griess reagent, and the absorbance was measured at 540 nm against a standard solution.

##### DPPH^•^ Radical-Scavenging Power

The capacity of *M. vulgare* to neutralize DPPH^•^ radicals was estimated based on the protocol of Yamaguchi et al. [79]. An amount of 1 mL of DPPH• reagent (0.1 mmol/L DPPH• in methanol) was add to 3 mL of each trial, mixed vigorously, and left to remain at room temperature for 30 min before a reading at 517 nm. The control was prepared using the same process but without the sample. The DPPH• radical content was calculated using the following formula:

*DPPH^•^ radical scavenging power* (%) = 100 − ((*A _control_* − *A _sample_*)/*A _control_* × 100)

##### ABTS Radical Cation Capture Power

The capacity of *M. vulgare* to capture the ABTS radical cations was assayed using the technique developed by Miller and Rice-Evans [80] and modified by Arnao et al. [81]. An amount of 0.2 mL of peroxidase (4.4 U/mL), 0.2 mL of H_2_O_2_ (50 μmol/L), 0.2 mL of ABTS (100 μmol/L), and 1 mL of methanol was added and kept in the dark for 1 h to form a bluish-green complex. Then, 1 mL of each sample was added to the complex, and the absorbance was measured at 734 nm. The ABTS radical cation capture power was calculated as follows:

*ABTS_•_ radical scavenging power* (%) = (1 − (*A _sample_*/*A _control_*)) × 100

##### H_2_O_2_ Scavenging Activity

The H_2_O_2_ scavenging ability of *M. vulgare* extract or the standard compounds were assessed using the method described by Ruch et al. [82]. Samples and standard concentrations were added to 0.6 mL H_2_O_2_ (40 mmol/L) and then read at 230 nm after 10 min against a phosphate buffer blank without H_2_O_2_. The H_2_O_2_ scavenging capacity was calculated as follows:

H_2_O_2_ % = *Inhibition* (%) = ((*A _control_* − *A _sample_*)/*A _control_*) × 100

#### 4.3.3. Anti-Inflammatory Activity Assay

##### Cyclooxygenase Inhibition Protocol

The cyclooxygenase reduction protocol was carried out based on the method of Larsen et al. [83], and celecoxib was employed as a reference drug. Leuco-2, 7-dichlorofluorescein diacetate (5 mg) was hydrolyzed at room temperature in 50 μL sodium hydroxide (NaOH, 1M) for 10 min, then 30 μL HCl (1M) was mixed to neutralize excess NaOH, before the resulting 1-DCF was diluted in 0.1 M Tris buffer, pH 8. The cyclooxygenase enzyme (COX-1 or COX-2) was diluted in 0.1 M Tris buffer, pH 8. The examined samples (20 μL) were incubated with the enzyme at room temperature for 5 min in the presence of hematin. To initiate the reaction, phenol (500 μM), 1-DCF (20 μM), and hematin (1 μM) were added to a 1 mL final volume of 0.1 M Tris buffer, pH8. The reaction was monitored for 1 min at 502 nm. The blank contained all of the reaction components except the enzyme.

#### 4.3.4. In Vitro Evaluation of the Anti-Alzheimer Effectiveness of the *M. vulgare* Extract

##### Inhibition of Acetylcholinesterase

The acetylcholinesterase activities (AChE and BChE) were analyzed based on the methods of Ingkaninan et al. [84]. The *M. vulgare* serial concentrations in methyl alcohol (100, 250, 500, 750, and 1000 μg/mL) were first organized. Also, the rest of the protocol steps were followed as described previously [15,84]. The measurement was conducted in triplicate, and the mean absorbance was used to calculate the inhibition percentage. The IC_50_ values were calculated using the log–probit analysis [15].

##### Inhibition of Tyrosinase

Liu et al. [85] conducted the tyrosinase inhibition assessment. The absorbance was calculated at 475 nm using a spectrophotometer. *M. vulgare* extract (40 μL) was liquefied in methanol and added to each well. Each well received 80 μL of phosphate buffer (pH 6.8), 40 μL of tyrosinase, and 40 μL of L-Dopa. The blank included all the constituents except L-Dopa. Kojic acid was considered a common inhibitor. The percentage of tyrosinase reduction in the extract and standard was determined three times [15].

### 4.4. Molecular Docking Study

To explore the molecular basis of the *M. vulgare* extract in combating Alzheimer’s disease, all 56 components identified in the extract were subjected to molecular docking studies with enzymes related to Alzheimer’s disease. As mentioned in a current report, docking experiments were designed by MOE software (Version 2014. 09, Chemical Computing Group Inc., Montreal, Canada) [86]. X-ray crystal structures of cyclooxygenase (PDB ID: 3mdl), acetylcholinesterase (PDB ID: 4ey7), butyrylcholinesterase (PDB ID: 1xlu), and tyrosinase (PDB ID: 5m8t) were restored from the Protein Data Bank (https://www.rcsb.org, access on 1 April 2024) and created with MOE. Water molecules located farther than 4.5 Å from the ligand or receptor were deleted, and structure preparation was performed to address protonation issues in the protein, using default settings in QuickPrep. The co-crystallized ligand was removed from the prepared protein and redocked into the binding site to validate the docking protocol by analyzing the RMSD and docking parameters. Both ligand and receptor molecules were energy-minimized using the Amber10:EHT force field. The Triangular Matching docking approach was used to dock all compounds into the active sites of the four enzymes. The number of docking iterations was set to 300, while the default values were used for the remaining parameters, resulting in five descriptions of each ligand–protein complex. The docking mode with the lowest energy was selected, and the docking findings were manually examined to determine the interactions of the drugs with binding pockets.

### 4.5. Statistical Analysis

The results are shown as mean ± SD. In vitro antioxidant data were analyzed using a one-way *t*-test (*n* = 4). The *p*-value was less than 0.05, indicating statistical significance.

## 5. Conclusions

In the current study, the crude extract of *M. vulgare* suppressed acetylcholinesterase and butyrylcholinesterase degradation, which are essential enzymes involved in correctly functioning processes related to memory and learning. It may also prevent the aggregation of neurofibrillary tangles by reducing tyrosinase activity. Additionally, *M. vulgare* has shown potential as a selective antioxidant and anti-inflammatory agent, which can prevent the cascade of amyloid-β and senile plaque formation. The antioxidant activity of *M. vulgare* can ultimately lead to the suppression of lipid peroxidation and the formation of amyloid-β. The current study effectively demonstrated the comprehensive role of the crude *M. vulgare* extract in treating Alzheimer’s syndrome according to its anti-acetylcholinesterase and anti-butyrylcholinesterase effects, anti-tyrosinase activities, and antioxidant and anti-inflammatory properties. In addition, the molecular docking studies introduced the phenylethanoid glycoside metabolites of marruboside, samioside, and forsythoside B as promising potential anti-Alzheimer agents with the lowest binding affinities towards the investigated enzymes.

## Figures and Tables

**Table 1:** The relative percentage of hydrocarbons and sterols identified by the GC-MS analysis of *M. vulgare* extract.

Peak	t_R_ (min)	Name	Formula	Chemical Class	Conc.
1	3.12	Desogestrel	C_22_H_30_O	Steroid	2.92
2	3.16	1-Heptatriacotanol	C_37_H_76_O	OHC	2.42
3	3.40	Cholest-22-ene-21-ol, 3,5-dehydro-6-methoxy-, pivalate	C_33_H_54_O_3_	Steroid	1.41
4	3.78	3-Ethyl-3-hydroxy-, (5.α.)-androstan-17-one	C_21_H_34_O_2_	Steroid	81.39
5	4.07	Ageratriol, trimethyl ether	C_18_H_30_O_3_	Sesquiterpene	2.60
6	4.37	Cholestan-3-ol, 2-methylene-, (3*β*, 5.α.)-	C_28_H_48_O	Steroid	3.97
7	6.62	Stigmasterol	C_29_H_48_O	Steroid	1.83
8	6.97	(3*β*, 24S)-Stigmast-5-en-3-ol	C_29_H_50_O	Steroid	2.05
9	7.83	Doconexent	C_22_H_32_O_2_	PUSFA	1.42

OHC: oxygenated hydrocarbons, PUSFA: polyunsaturated fatty acid, t_R_: retention time, Conc.: concentration, % = % of individual peak area/total sum of peak areas × 100.

**Table 2:** Chemical compositions of saponifiable matter of *M. vulgare* identified by GC-MS analysis.

Peak	t_R_ (min)	Name	Chemical Formula	Conc. %
1	11.269	Benzoic acid, 2-ethylhexyl ester	C_15_H_22_O_2_	0.11
2	11.618	Hexadecanoic acid, methyl ester	C_17_H_34_O_2_	1.94
3	13.473	Methyl stearate	C_19_H_38_O_2_	0.27
4	13.682	9-Octadecenoic acid (*Z*)-, methyl ester	C_19_H_36_O_2_	0.27
5	14.256	9,12-Octadecadienoic acid (*Z*, *Z*)-, methyl ester	C_19_H_34_O_2_	0.56
6	14.633	Methyl 5,6-octadecadienoate	C_19_H_34_O_2_	0.34
7	15.188	9,12,15-Octadecatrienoic acid, methyl ester, (*Z*,*Z*,*Z*)-	C_19_H_32_O_2_	0.33
8	16.625	17-Octadecynoic acid, methyl ester	C_19_H_34_O_2_	0.06
9	16.778	Methyl 9-cis,11-trans-octadecadienoate	C_19_H_34_O_2_	0.06
10	19.038	Methyl 2-ethylhexyl phthalate	C_17_H_24_O_4_	2.95
11	19.392	Phthalic acid, cyclohexyl methyl ethyl ester	C_17_H_22_O_4_	0.08
12	29.567	Phthalic acid, di(2-propylpentyl) ester	C_24_H_38_O_4_	92.25
13	33.184	Decanedioic acid, bis(2-ethylhexyl) ester	C_26_H_50_O_4_	0.24
14	34.048	Octadecanoic acid, 9,10-dihydroxy-, methyl ester	C_19_H_38_O_4_	0.14
15	35.18	1,3-Benzenedicarboxylic acid, bis(2-ethylhexyl) ester	C_24_H_38_O_4_	0.4
		Sum		100
		SAT. FAME		2.59
		UNSAT. SFAME		1.62
		Benzoic acid derivatives		95.79

Retention time (t_R_), SAT: saturated, UNSAT: unsaturated; FAME: fatty acid methyl ester.

**Table 3:** Identified compounds of *M. vulgare* butanol fraction using LC/MS^n^.

Peak	t_R_ (min)	Identified Metabolites	[M − H]^−^	Ms/Ms
1	1.7	Quinic acid ^#^	191	173, 127, 111
2	3.13	Aconitic acid ^#^	173	85, 129
3	4.19	Methyl gallic acid ^#^	183	169, 139, 125
4	4.99	Malic acid ^#^	133	115, 87, 71
5	5.93	Apigenin-7-*O*-xyloside	401	287, 269, 221, 219,113
6	6.6	Protocatechuic acid ^#^	153	109
7	7.41	Cinnamic acid	147	103, 77
8	7.73	Ferulic acid	193	134
9	8.35	Caffeic acid ^#^	179	135
10	8.90	4-hydroxy benzoic acid ^#^	137	93, 65
11	9.55	Gallic acid ^#^	169	125
12	10.50	Vanillin ^#^	151	136, 135, 92
13	10.88	Vitexin “ Apigenin-8-*C*-glucoside” *	431	341, 311, 283, 269
14	12.631	Catechin	289	245, 221, 109
15	12.65	Apigenin-7-*O*-xyloside	401	287, 269, 221, 219, 113
16	12.97	*p*-Coumaric acid ^#^	163	119, 93
17	14.67	Quercetin *	301	273, 257
18	14.75	Naringenin ^#^	271	151, 119
19	15.13	Apigenin-7-*O*-xyloside	401	287, 269, 221, 219, 113
20	15.15	Apigenin *	269	151, 117
21	25.24	luteolin-7-glucoside *	447	285
22	25.67	Luteolin *	285	151, 133
23	28.17	luteolin-7-*O*-lactate *	357	285, 269, 223
24	29.4	Chrysoeriol *	299	285, 284, 269
25	32.2	Apigenin *	269	151, 117
26	35.14	Chrysin ^#^	253	143, 119

t_R_ = retention time: [M − H]^−^ = precursor mass; ^#^ identified previously from the same genus [27], * isolated previously [28].

**Table 4:** Identified phenylethanoid glycoside and high (*O* and *C*) glycoside compounds of *M. vulgare* aqueous fraction using LC/MS^n^.

Peak	t_R_ (min)	Identified Metabolites	[M − H]^−^	Ms/Ms
1	6.36	Marruboside ^ 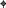 ^	887	725, 593
2	6.51	Verbascoside ^ 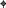 ^	623	461, 315
3	6.80	Forsythoside B ^ 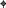 ^	755	593, 461, 447
4	7.00	Samioside ^ 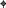 ^	755	593, 461
5	7.45	Apigenin-7-*O*-neohesperidoside ^#^	577	431, 269
6	8.00	Apigenin-7-*O*-diglucuronide-*O*-hexoside	783	737, 607, 431, 269
7	8.59	Apigenin-7-*O*-diglucuronide-*O*-hexoside	783	737, 607, 431, 269
8	9.00	Isoscutellarein-7-*O*-(6-*O*-acetylallosyl) glucoside ^#^	651	591, 489, 285, 257, 217, 175
9	9.49	Apigenin-7-*O*-neohesperidoside ^#^	577	431, 269
10	10.45	Vicenin II (apigenin 6,8-di-*C*-glycoside) ^#^	593	503, 383
11	14.82	Marrubiin ^ 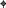 ^	331	313, 303, 287, 285

^
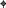
^ Identified previously from the same species [29], ^#^ identified previously from the same genus [30,31].

## Data Availability

Data is contained within the article or Supplementary Material.

## Supplementary Materials

The following supporting information can be downloaded at: https://www.mdpi.com/article/10.3390/ph17101282/s1, Table S1: molecular docking results.
